# Ascorbic acid induced TET2 enzyme activation enhances cancer immunotherapy efficacy in renal cell carcinoma

**DOI:** 10.7150/ijbs.67329

**Published:** 2022-01-01

**Authors:** Ding Peng, Anbang He, Shiming He, Guangzhe Ge, Shuo Wang, Weimin Ci, Xuesong Li, Dan Xia, Liqun Zhou

**Affiliations:** 1 Department of Urology, Peking University First Hospital, No. 8 Xishiku Street, Beijing 100034, P.R. China.; 2 Department of Urology, The First Affiliated Hospital School of Medicine, Zhejiang University, 79 Qingchun Road, Hangzhou, Zhejiang Province 310003, P.R. China.; 3 Key Laboratory of Genomics and Precision Medicine, Beijing Institute of Genomics, Chinese Academy of Sciences, No.1 Beichen West Road, Beijing,100101, P.R. China.

**Keywords:** Immunotherapy, DNA hydroxymethylation, Renal cell carcinoma, Ascorbic acid, TET2

## Abstract

Exploring the regulatory mechanism of PD-L1 in renal cancer is one of the key strategies to improve the response of renal cancer patients to checkpoint blockade therapy. In this study, the synergistic effect of ascorbic acid (vitamin C) supplementation and the impact of TET2 depletion on anti-PD-L1 therapy were determined in xenograft model experiments. Lymphocyte infiltration and chemokine expression were determined using flow cytometry and qRT-PCR. To determine the downstream targets of TET2, we performed hMeDip-seq and RNA-seq analyses. The molecular mechanism was further confirmed by hMeDip-qPCR, MeDip-qPCR, bisulfite sequencing, Western blotting, qRT-PCR and xenograft model experiments *in vitro* and *in vivo*. The present study demonstrated that ascorbic acid enhanced the efficacy of immunotherapy and that the loss of TET2 function enabled renal cancer cells to evade antitumor immunity. Ascorbic acid treatment significantly increased the intratumoral infiltration of T cells and the expression of cytokines and chemokines, while the loss of TET2 impaired the infiltration of T cells and the expression of cytokines and chemokines. TET2 was recruited to IRF1 by IFN-γ-STAT1 signaling, thereby maintaining IRF1 demethylation and ultimately inducing PD-L1 expression. These results suggest a new strategy of stimulating TET activity to improve immunotherapy for renal cell carcinoma.

## Introduction

Renal cell carcinoma (RCC) is one of the most common genitourinary cancers and accounts for 2% to 3% of all malignant cancer cases 1, 2. Clear cell renal cell carcinoma (ccRCC) is the most common subtype and accounts for more than 80% of renal cancers. Approximately 20% of RCC patients are already of advanced stage at the time of diagnosis, while nearly 30% of local RCC patients will develop recurrence or metastasis after surgical resection 3, 4.

Immunotherapy for the treatment of RCC began approximately 30 years ago, and high-dose interleukin 2 (IL-2) still represents an effective therapy with a lasting clinical response 5. In the past few years, cancer immunotherapy has made significant progress in RCC. Immune checkpoint blockade is a new type of immunotherapy that reduces inhibitory signal transduction and restores tumor-specific T cell-mediated immune responses. Immune checkpoints, such as cytotoxic T lymphocyte-associated antigen (CTLA-4) and programmed death ligand 1 (PD-L1), inhibit the activity of T lymphocytes to identify and eliminate cancer antigens 6. Activation of the PD-1/PD-L1 pathway is one of the main mechanisms of tumor immune escape, which inhibits the proliferation and activation of T cells, ultimately leading to the immune escape of tumor cells and induction of tumor development and metastasis 7. Therefore, PD-1/PD-L1 is the most promising target for cancer immunotherapy, including RCC. Recently, several anti-PD-1/PD-L1 drugs have been approved for the treatment of advanced RCC and have shown acceptable efficacy 5, 8. However, due to the difference in the expression of PD-1/PD-L1 and its diverse regulatory mechanisms, drug sensitivity to anti-PD-1/PD-L1 varies greatly due to individual differences. For all the above reasons, a safe combination strategy needs to be determined to enhance tumor sensitivity to these drugs and to expand the number of RCC patients benefiting from cancer immunotherapy.

Epigenetic targeting agents, including DNA methyltransferase inhibitors (DNMTIs), in combination with immune checkpoint blockade therapies have been explored, and have been shown to exert synergistic effects 9, 10. 5-Hydroxymethylcytosine (5hmC) is catalyzed from 5mC by TET enzymes in the demethylation cycle and has been found to be a conversion form of 5mC 11. In many kinds of cancers, 5hmC levels have been found to be significantly reduced and associated with tumorigenesis, progression and outcomes 12-16. Some studies, including our previous work, have shown that 5hmC is significantly lost in renal cell carcinoma and that ascorbic acid (vitamin C) treatment restores the 5hmC pattern of renal cell carcinoma by stimulating the activity of the TET2 enzyme, thereby changing the epigenome and transcriptome of renal cancer cells and inhibiting the related malignant phenotype 13, 17, 18. Recent studies have also shown that high-dose vitamin C regulates the infiltration of immune cells into the tumor microenvironment and delays the growth of cancer cells *in vivo* in a T-cell-dependent manner 19, 20. Vitamin C enhances the cytotoxic activity of CD8+ T cells, and it is also used in conjunction with immune checkpoint therapy (ICT) for various cancer types 19, 20. It has also been reported that TET enzymes mediate the IFN-γ/JAK/STAT signaling pathway to control the expression of chemokines, lymphocyte infiltration and cancer immunity 21-23. Therefore, we speculated that restoring 5hmC levels in RCC promoting TET2 activity may have a synergistic effect with immune checkpoint therapy.

The present study showed that stimulating TET2 activity by systematic injection of vitamin C hypomethylates IRF1 to restore PD-L1 levels, leading to enhanced antitumor immunity and anti-PD-L1 efficacy. Our findings provide compelling evidence to test the combination of high-dose vitamin C and immune checkpoint therapy in patients with renal cell carcinoma.

## Materials and Methods

### Cell culture and reagents

Renca, 786-O and A498 cells were cultured in high-glucose DMEM containing 10% fetal bovine serum and 1% penicillin/streptomycin. The following antibodies were used: anti-5hmC antibody (Active Motif, 39769), anti-5mC antibody (ZYMO, A3001-200), IRF1 (Abcam, ab232861), TET2 (Abcam, ab9458), beta-tubulin (Abcam, ab179511), p-STAT1 (Cell Signaling Technology, 9167), PD-L1 (Cell Signaling Technology, 13684), CD4 (Abcam, ab237722) and CD8 (Abcam, ab217344).

### Generation of the TET2 knockout cell line

TET2 knockout Renca, 786-O and A498 cell lines were established using the CRISPR/Cas9 system as described in a previous study 17. The oligonucleotide encoding sgRNA was cloned into the BsmBI-digested pLenti-CRISPRv2 vector, and lentivirus was generated. Cells infected with lentivirus were selected, and the TET2 knockout efficiency was evaluated by Western blot analysis. The knockout cell pools were inoculated in 10-cm culture dishes, and single clones were selected. The knockout efficiency of TET2 in each clone was measured by Western blotting.

### MTS cell viability assay

The cell viability assays were assessed using CellTiter 96® AQ One Solution Reagent (Promega, USA) according to the manufacturer's instructions.

### Dot blot

Genomic DNA was extracted from the cultured cells according to the manufacturer's instructions. The DNA samples were diluted with 2 M NaOH and 10 mM Tris·Cl (pH 8.5), and they were then loaded onto Hybond N^+^ nylon membranes using a 96-well dot blot apparatus. After incubation at 80°C for 60 minutes and blocking with 5% skimmed milk at room temperature for 1 hour, the membrane was incubated with the anti-5hmC antibody at 4°C overnight and visualized by chemiluminescence.

### qRT-PCR

Total RNA was isolated from cell lines using TRIzol reagent. A total of 2 μg of RNA was reverse-transcribed into cDNA using M-MLV reverse transcriptase. Quantitative PCR was performed using SYBR® FAST qPCR Kits with a final volume of 10 μl and the 7500 Fast Real-Time PCR System. The expression of target mRNA was normalized to GAPDH.

### DNA bisulfite treatment and methylation analysis

For the bisulfite DNA methylation analysis, 1 μg of genomic DNA was denatured using 0.3 M NaOH for 10 min at 37°C. After adding hydroquinone and sodium bisulfate, the samples were incubated at 50°C for 16 hours. For the BSP analysis, the DNA was purified, and the CpG-rich promoter region was amplified by PCR. The primer sequences were as follows: IRF1-F, 5'-GTTAGGGGTTTGTAGGTGGT-3'; and IRF1-R, 5'-ATTTCCCCTCCTCTAAAAAAAT-3'. The PCR products were purified and cloned into the PCR 2.1-TA cloning vector. At least ten positive clones from each product were selected for sequencing.

### Western blot analysis

Total protein from cells was isolated using ice-cold radioimmunoprecipitation assay buffer and quantified with BCA protein assay reagent. The same amount of protein was then separated by SDS-PAGE and transferred to a polyvinylidene fluoride membrane. After blocking with 5% milk for 1 hour, the membrane was incubated with a primary antibody overnight at 4°C. After washing and incubating with the secondary antibody, the signal was detected using ECL Western blotting detection reagent.

### Mouse xenograft model experiment

For the mouse xenograft experiment, 786-O and A498 cells were suspended in 100 µl of Matrigel matrix diluted with PBS at 1:1 and subcutaneously injected into the axillary fossae in BALB/c nude mice. Renca cells were suspended in 100 µl of Matrigel matrix diluted with PBS at 1:1 and subcutaneously injected into the axillary fossae of BALB/c mice. Tumor volume was measured once tumors were palpable and continued through the end of the study. Tumor volume was calculated with the following formula: V = 0.5×a×b^2^; where a is the longest tumor axis; and b is the shortest tumor axis. For the vitamin C treatment model, mice were intraperitoneally injected with 0.5 g/kg ascorbic acid every day. For the anti-PD-L1 immunotherapy model, mice were intraperitoneally injected with 200 μg of anti-PD-L1 antibody on alternating days. The animal protocol was approved by the Animal Ethics Committee of Peking University First Hospital.

The coefficient of drug interaction (CDI) was calculated to test for synergy between vitamin C and anti-PD-L1 using mean tumor weight measurements (with CDI < 0.7 indicating a significantly synergistic effect) as follows:



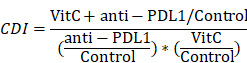



### Flow Cytometry

Mouse tumors were cut into small pieces, disaggregated with collagenase and filtered through 70-μm strainers. For membrane staining, cells were incubated with purified antibodies for 30 min at 4°C. In a coculture system, staining was performed according to the manufacturer's instructions. Stained samples were detected and analyzed by eight-color flow cytometry (BD Biosciences, FACSC alibur).

### hMeDIP-seq

The sequencing libraries were prepared with 10 μg of genomic DNA and ligated to PE adaptors (Illumina) followed by 5hmC antibody capture for immunoprecipitation. The hydroxymethylated fragments were amplified with 10-12 cycles using adaptor-specific primers (Illumina) and quantified on an Agilent 2100 Bioanalyzer before cluster generation and sequencing on a HiSeq 3000 according to the manufacturer's protocols.

### Identification of 5hmC peaks

Briefly, the reads were aligned to the hg19 human genome and deduplicated; unique reads were kept. Significantly enriched regions were determined using model-based analysis with the ChIP-Seq (MACS) package (v.2.1.0, default settings).

### hMeDIP-qPCR and MeDIP-qPCR

Genomic DNA (2 μg) was sonicated by a Covaris Focused-ultrasonicator and then heat denatured, and 5% of the DNA was reserved as the input. The remaining portion was divided equally, and 0.5 μl of 5hmC antibody or 3 μg of 5mC antibody was added and incubated at 4°C overnight. Protein G beads were added to capture the complexes, and the eluted DNA was subsequently used for qPCR analysis. The primer sequences were as follows: IRF1-F, 5'- GTAGGTGGCCCGCTATGTT-3'; and IRF1-R, 5'- TAGGTCACGATTCCCTCCAG-3'.

### RNA-seq and data analysis

The KAPA Stranded RNA-seq Library Preparation Kit was used to construct RNA-seq libraries according to the manufacturer's instructions. Sequencing reads were aligned to the hg19 human genome using the TopHat program (TopHat v2.1.1) with the default parameters. Total read counts for each protein-coding gene were extracted using HTSeq (HTSeq version 0.6.0) and then loaded into the R package DEseq2 to calculate the differentially expressed genes with FDR < 0.05.

### Statistical analysis

All statistical analyses had at least three independent replicates. Data are represented as the mean ± SD and were analyzed by GraphPad Prism. Statistical significance was determined by a two-tailed Student's t-test for two group comparisons. Significance in all figures is indicated as follows: NS > 0.05, *P < 0.05, **P < 0.01 and ***P < 0.001.

## Results

### Vitamin C sensitizes renal cell carcinoma to anti-PD-L1 treatment

**Figure 1:**
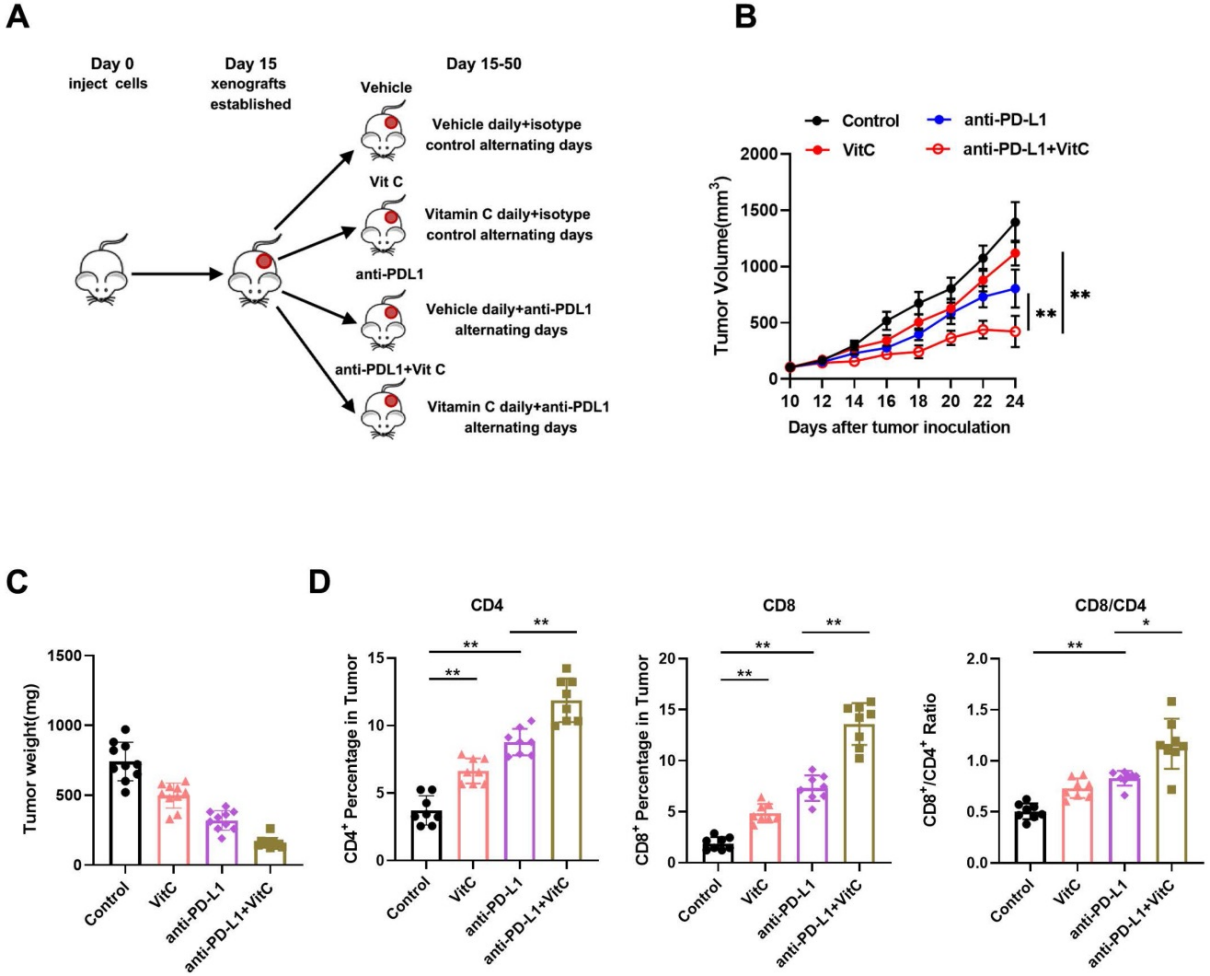
High-dose vitamin C treatment synergizes with anti-PD-L1 checkpoint inhibition in a renal cell carcinoma mouse model. (A) BALB/c mice received injection of Renca cells and were randomly assigned to 4 groups as follows: vehicle, anti-PD-L1, vitamin C and vitamin C+anti-PD-L1. (B) The survival curve of mice in different groups (n = 10 mice for each group). (C) Tumor weights of mice in different groups. (D) Percentage of CD4+ and CD8+ T cells as well as the CD8+/CD4+ ratio in tumors in (B). *P < 0.05, **P < 0.01, ***P < 0.001.

First, we investigated the synergistic effect of vitamin C on anti-PD-L1 therapy *in vivo*. We treated tumor-bearing Renca xenograft model mice with vehicle, anti-PD-L1, high-dose vitamin C or the combination of high-dose vitamin C and anti-PD-L1 (Fig. 1A). The results showed that the single agent anti-PD-L1 or single agent vitamin C did demonstrate a trend toward proliferation inhibition compared to the vehicle group (Fig. S1A). Moreover, the growth curve of the vitamin C+anti-PD-L1 group was significantly divergent from that of the vehicle group and single agent groups (Fig. 1B). The coefficient of drug interaction (CDI) between high-dose vitamin C and anti-PD-1 using the mean tumor weight measurements was 0.61, indicating a significantly synergistic effect (CDI < 0.7) (Fig. 1C).

In support of the immunomodulatory function of vitamin C, flow cytometry analysis of tumors showed that vitamin C treatment induced tumor infiltration by both CD4+ and CD8+ T lymphocytes and increased the CD8+/CD4+ ratio. Similarly, injection of anti-PD-L1 antibody resulted in a significant increase in intratumoral CD4+ and CD8+ T cells. Moreover, the intratumoral CD4+ and CD8+ T lymphocyte fractions and the CD8+/CD4+ ratio were further increased by combining vitamin C and anti-PD-L1 therapy (Fig. 1D and Fig. S1B). These results indicated that the addition of vitamin C to anti-PD-L1 therapy enhances the recruitment of T lymphocytes in the tumor microenvironment. The above results led us to determine the expression of PD-L1 and the IFN-γ-induced chemokines, CXCL9, CXCL10 and CXCL11. We found that the expression of PD-L1, CXCL9, CXCL10 and CXCL11 was significantly increased in vitamin C-treated tumors compared to that in control tumors (Fig. S1C). Importantly, vitamin C treatment increased the expression of PD-L1, CXCL9, CXCL10 and CXCL11 in 786-O and Renca cells in response to IFN-γ stimulation (Fig. S1D). Together, these results indicate a mechanism by which vitamin C enhances the efficacy of immunotherapy by modulating the production of cytokines and chemokines in the tumor microenvironment.

### Loss of TET2 confers renal cell carcinoma resistance to immunotherapy

**Figure 2:**
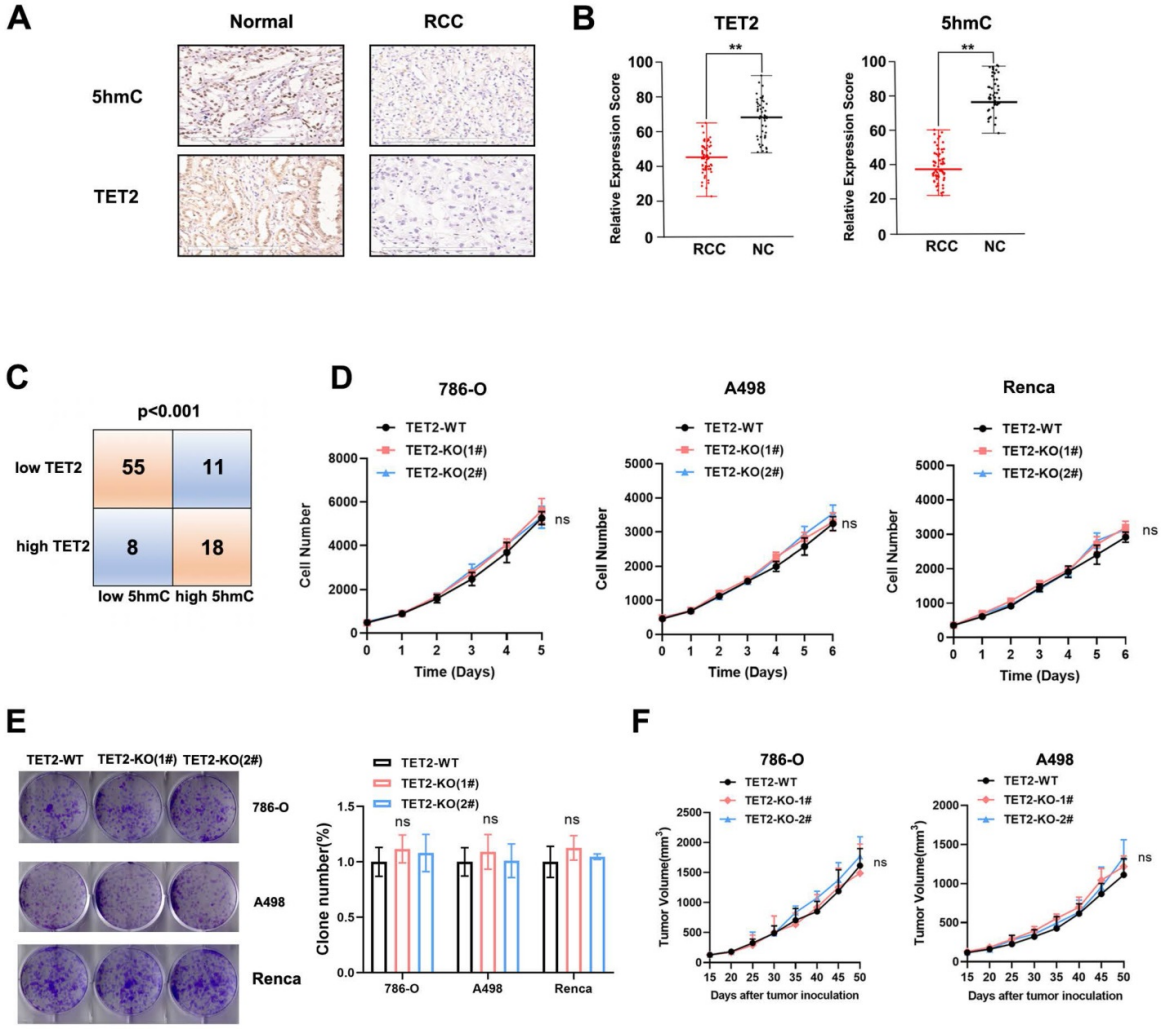
Low TET2 expression reduces 5hmC levels and does not affect cell proliferation. (A) Representative images of immunohistochemical analysis of TET2 and 5hmC in ccRCC samples and normal kidney tissues. (B) Quantification of TET2 and 5hmC expression in ccRCC samples and normal kidney tissues. (C) Correlation analysis of TET2 and 5hmC expression levels. (D) MTS assay of TET2-WT and TET2-KO cell viability. (E) Colony formation assay for TET2-KO and TET2-WT cells. (F) Tumor growth curves of xenografts with TET2-WT and TET2-KO cells. *P < 0.05, **P < 0.01, ***P < 0.001.

Vitamin C is reported to be a cofactor for TET enzymes by promoting the recycling of inactive oxidized ferric iron (Fe^3^+) to actively reduced ferrous iron (Fe^2^+) as well as TET protein folding, and it has been shown to stimulate TET activity* in vitro* and *in vivo*^24^-^26^. Our previous study showed that the expression of TET2 is the highest among the TET genes in RCC^17^. Therefore, we measured TET2 expression and 5hmC levels in ccRCC samples by IHC (Fig. 2A). The results indicated significantly reduced TET2 expression and 5hmC levels in ccRCC samples compared to their normal counterparts (Fig. 2B). Further analysis showed that low TET2 expression was significantly associated with low 5hmC levels (Fig. 2C).

To further explore the function of the TET2 enzyme in the immunotherapy effect in RCC, we next deleted TET2 using CRISPR/Cas9 genome-editing technology in 786-O, A498 and Renca cells (Fig. S2A). Dot blot staining showed that the 5hmC levels were significantly decreased in TET2-KO cells, and the addition of vitamin C stimulated TET activity significantly in TET2-WT cells but only minimally in TET2-KO cells (Fig. S2B). We then assessed whether TET2 depletion affects cell or tumor growth. TET2-KO 786-O, A498 and Renca cells showed rates of proliferation and colony formation similar to those of TET2-WT cells (Fig. 2D and Fig. 2E). Moreover, TET2-KO 786-O and A498 cells showed similar tumor growth in the nude mouse model compared to the parental TET2-WT cells (Fig. 2F). Collectively, these results suggested that TET2 does not play a significant intrinsic role in RCC cell proliferation* in vitro* or tumor growth *in vivo*.

**Figure 3:**
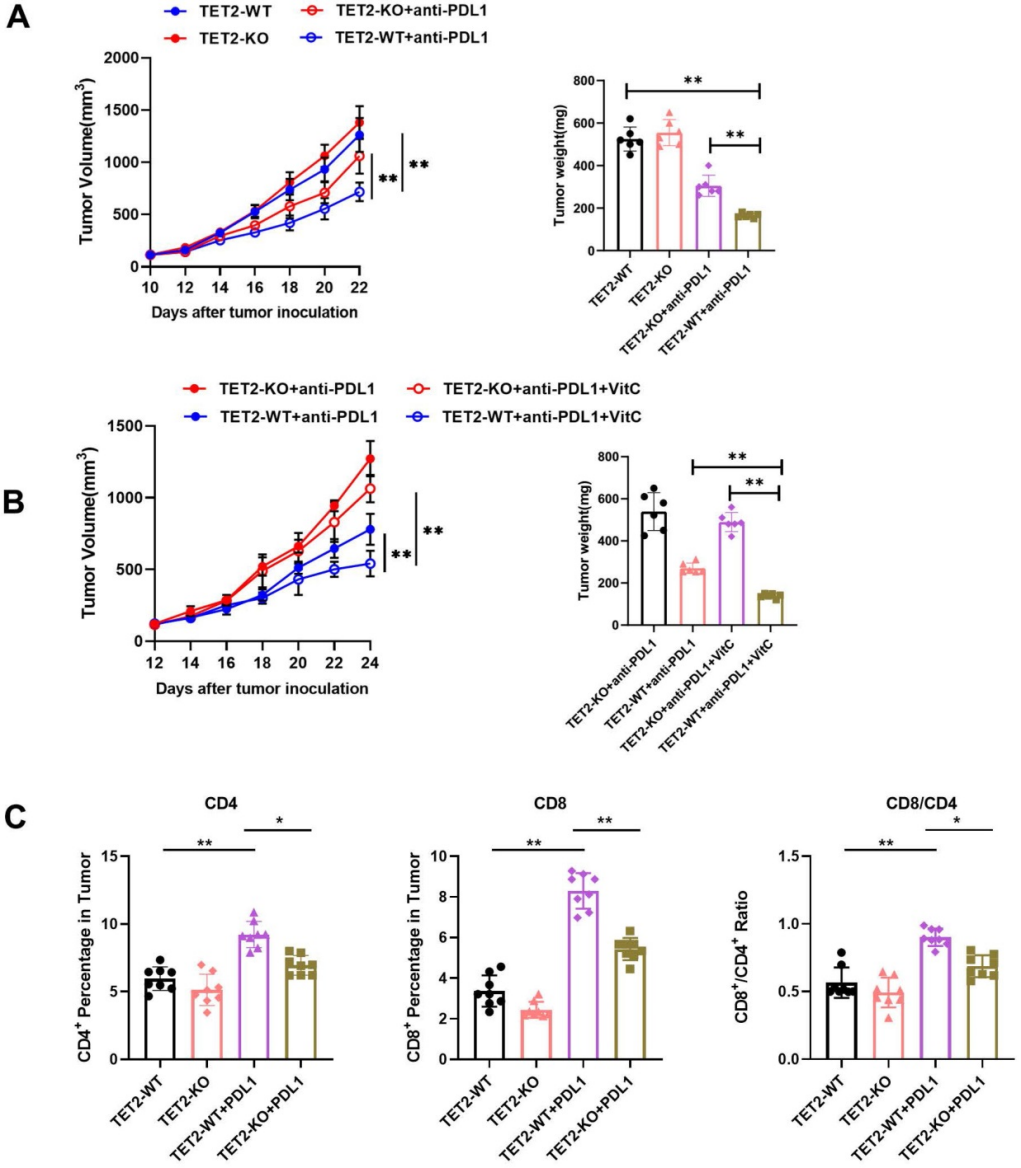
Loss of TET2 confers tumor resistance to anti-PD-L1 immunotherapy. (A) Tumor growth curves and tumor weights for mice injected with TET2-WT or TET2-KO Renca cells and treated with anti-PD-L1 antibody. (B) Tumor growth curves and tumor weights for mice injected with TET2-WT or TET2-KO Renca cells and treated with anti-PD-L1 antibody and vitamin C. (C) CD4+ and CD8+ T cell tumor infiltration and the CD8+/CD4+ ratio detected from (A). *P < 0.05, **P < 0.01, ***P < 0.001.

Next, to determine the role of TET2 in the response to immunotherapy, mice that received subcutaneous transplantation of TET2-WT and TET2-KO Renca cells were treated with an anti-PD-L1 antibody. We found that anti-PD-L1 treatment resulted in less inhibition of TET2-KO tumors than TET2-WT tumors (Fig. 3A). Furthermore, vitamin C injection conferred significantly more benefits to mice bearing TET2-WT tumors with slower tumor growth than mice with TET2-KO tumors as an adjuvant for anti-PD-L1 immunotherapy (Fig. 3B). These findings indicated that TET2 loss impairs the efficacy of anti-PD-L1 immunotherapy. Compared to TET2-WT tumors, TET2-KO tumors without injection of anti-PD-L1 antibody had significantly fewer CD4+ and CD8+ T cells as well as a decreased CD8+/CD4+ ratio. Injection of anti-PD-L1 antibody also resulted in a significant increase in intratumoral CD8+ and CD4+ T cells as well as an increased CD8+/CD4+ ratio in TET2-WT tumors but only a slight increase in TET2-KO tumors (Fig. 3C and Fig. S3A). These results suggested that loss of TET2 reduces the infiltration of T cells, leading to decreased antitumor immunity and induced resistance to anti-PD-L1 immunotherapy. The expression of PD-L1, CXCL9, CXCL10 and CXCL11 was also found to be significantly decreased in TET2-KO tumors compared to TET2-WT tumors (Fig. S3B). We then treated 786-O, A498 and Renca cells with IFN-γ and found that IFN-γ potently induced the expression of the PD-L1, CXCL9, CXCL10 and CXCL11 genes and that deletion of TET2 significantly reduced the IFN-γ induction of these genes (Fig S4A). These results demonstrated that loss of TET2 impairs IFN-γ-induced chemokine and PD-L1 expression as well as infiltrating lymphocytes in RCC.

### Identification of potential targets of TET2 in immunoregulation

**Figure 4:**
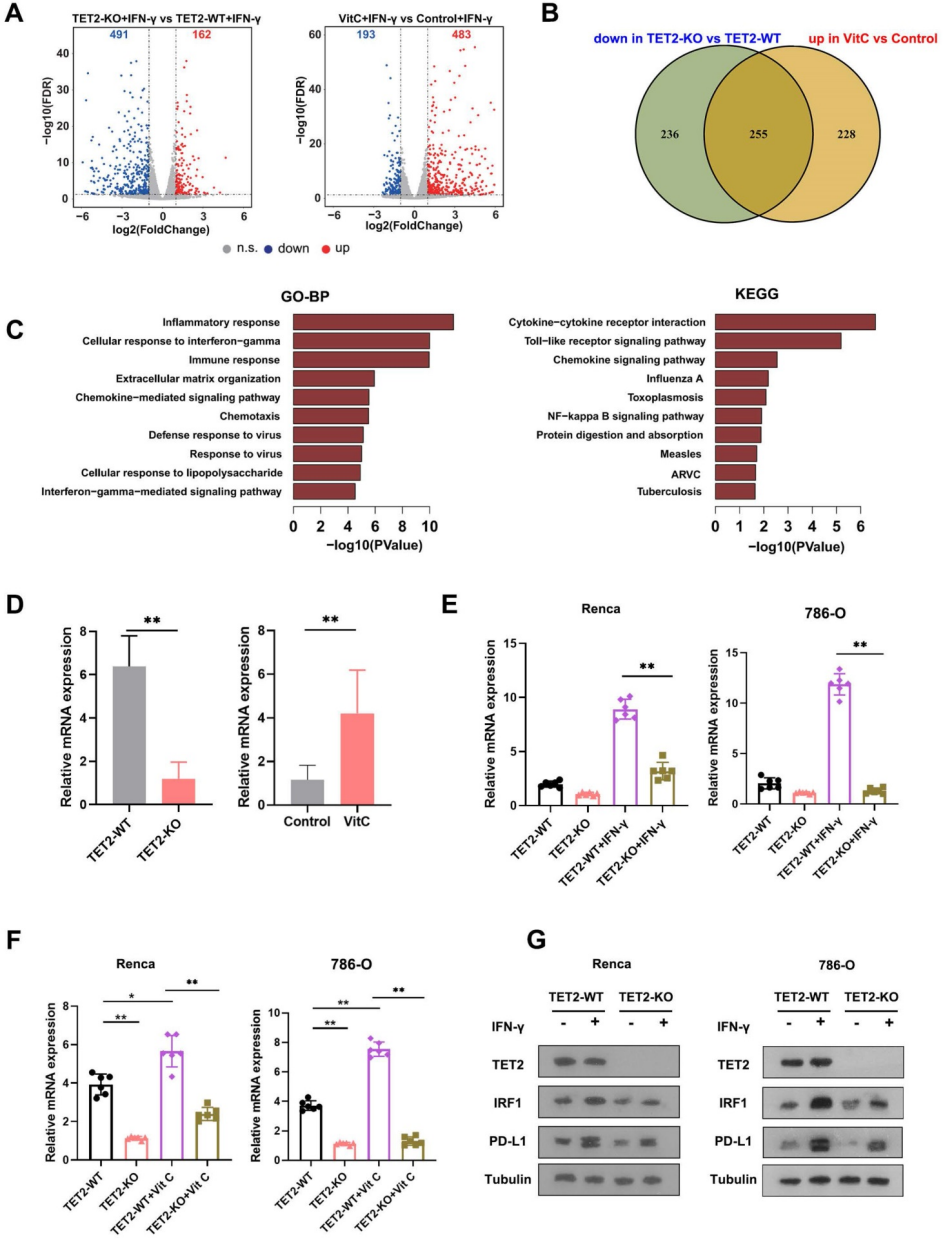
Identification of target genes of TET2 through 5hmC with RNA-seq and hMedip-seq. (A) Volcano plot of differentially expressed genes in TET2-KO and vitamin C-treated cells compared to control cells after IFN-γ stimulation. (B) Venn diagrams showing 255 significantly coregulated genes in the indicated cells. (C) GO and KEGG enrichment analysis for 255 significantly coregulated genes. (D) Relative mRNA levels of IRF1 determined by qRT-PCR in TET2-KO and vitamin C-treated tumors compared to control tumors. (E) Relative mRNA levels of IRF1 determined by qRT-PCR in TET2-WT or TET2-KO cells treated with IFN-γ. (F) Relative mRNA levels of IRF1 determined by qRT-PCR in TET2-WT or TET2-KO cells treated with vitamin C. (G) Western blot assay of the protein levels of TET2, IRF1 and PD-L1 in TET2-WT and TET2-KO cells with or without IFN-γ stimulation. *P < 0.05, **P < 0.01, ***P < 0.001.

To understand the molecular mechanism of TET2 in cancer immunotherapy, we employed RNA sequencing (RNA-seq) to identify the impact of vitamin C treatment and TET2 depletion on the transcriptome of RCC cells. Through RNA-seq analysis, we identified that 162 genes were upregulated and 491 genes were downregulated in TET2-KO cells compared to TET2-WT cells, and we also found that 483 genes were increased and 193 genes were decreased in vitamin C-treated cells compared to the control after IFN-γ stimulation (Fig. 4A). Furthermore, 255 common genes were identified that were downregulated in TET2-KO cells and increased in vitamin C-treated cells (Fig. 4B, Table S1). Gene ontology (GO) and KEGG analyses were performed on the 255 co-regulated genes, and these enriched pathways were mainly associated with responses to interferons, inflammation and cytokine production (Fig. 4C). Notably, vitamin C- and TET2-dependent genes were involved in interferon-gamma and interferon-beta pathways, including STAT1, IRF1, IRF7 and PD-L1, as well as cytokine-/chemokine-mediated signaling pathways, such as CCL3, CCL4, CCL5, CXCL9, CXCL10 and CXCL11, which was consistent with our previous observation of the production of chemokines. To validate these results, we performed qRT-PCR, and the results showed that all of these genes involved in interferon and cytokine/chemokine pathways were significantly upregulated in vitamin C-treated cells and decreased in TET2-KO cells (Fig. S4B). Together, these findings suggested that the co-regulated genes upon vitamin C treatment and TET2 depletion are principally connected with immune response-associated processes.

**Figure 5:**
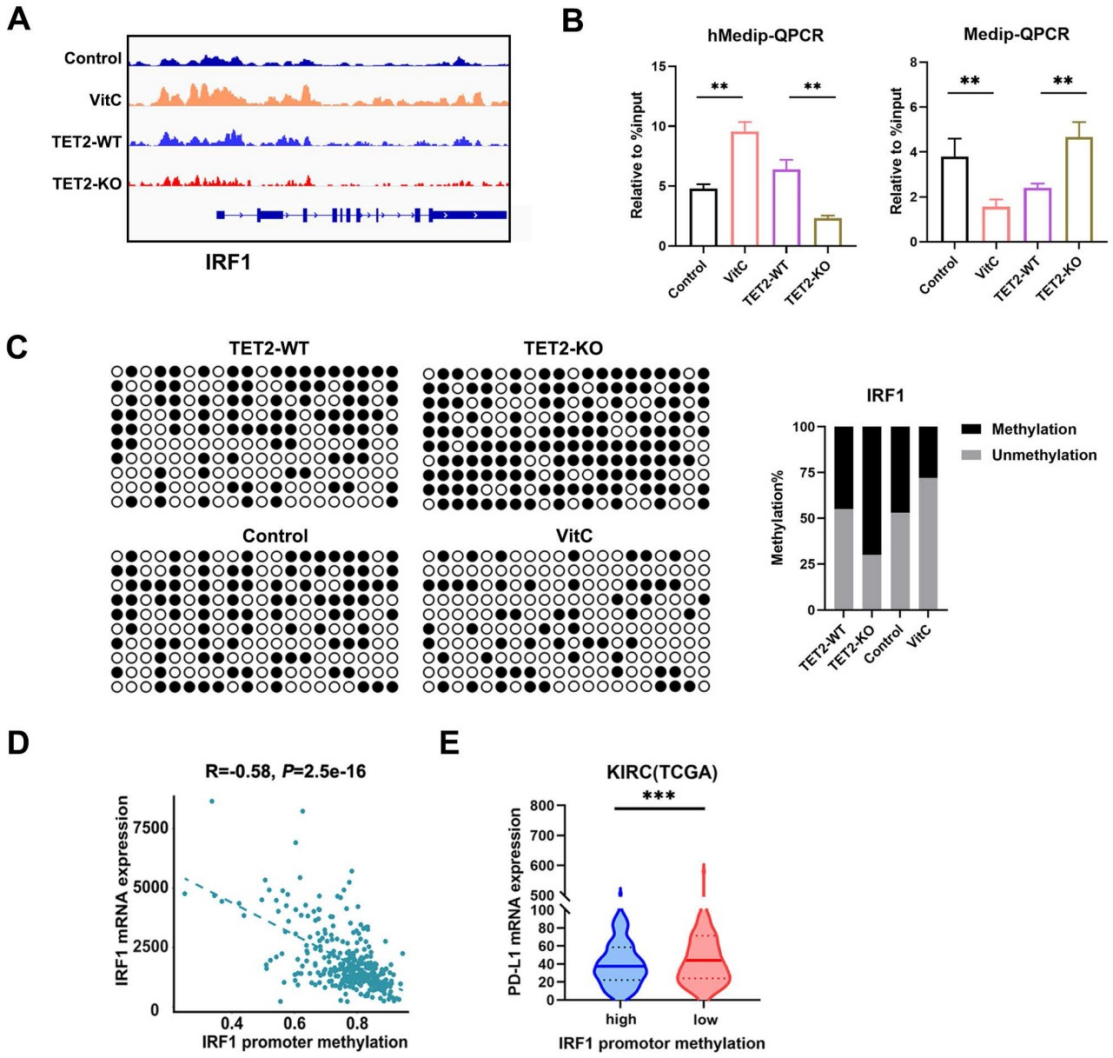
Vitamin C stimulates TET2 activity to hypomethylate and upregulate the expression of IRF1. (A) Representative 5hmC sites in IRF1 genes represented by integrative genomics viewer. (B) 5hmC and 5mC changes in IRF1 measured by hMeDIP-qPCR and MeDip-qPCR. (C) Bisulfite genomic sequencing analysis of the DNA methylation status of the IRF1 promoter. (D) The correlation of promoter methylation level and mRNA expression of IRF1 in ccRCC samples from TCGA dataset. (E) Correlation of IRF1 methylation and PD-L1 expression in human ccRCC tissues from TCGA dataset. *P < 0.05, **P < 0.01, ***P < 0.001.

To further identify the potential targets of TET2 through 5hmC, we next applied hMedip-seq analysis to TET2-KO and vitamin C-treated cells as described previously 17, 27. The analysis showed that the global genomic 5hmC levels were reduced after TET2 knockdown and increased after vitamin C treatment (Fig. S4C and S4D). We identified 2131 overlapping genes with significant 5hmC peaks in TET2-WT and vitamin C-treated cells. We next overlapped hMedip-seq analysis results and RNA-seq analysis results and identified 42 candidate genes, including IRF1 (Table S2). We then detected the mRNA levels of IRF1 in TET2-KO and vitamin C treated tumors compared to control tumors and found that the expression of IRF1 was significantly reduced in TET2-KO tumors but increased in vitamin C treated tumors compared to control tumors (Fig. 4D). In addition, deletion of TET2 significantly reduced the effect of IFN-γ and vitamin C on IRF1 induction (Fig. 4E and 4F). Western blot assays also showed that TET2 deletion suppressed the expression of IRF1 and PD-L1 (Fig. 4G). Importantly, the 5hmC levels in the promoter of IRF1 were significantly decreased after TET2 KO and significantly increased after vitamin C treatment, suggesting that TET2 may mediate PD-L1 expression by regulating the demethylation of IRF1 (Fig. 5A).

### TET2 regulates PD-L1 expression through IRF1 demethylation

IRF1 not only plays a basic role in JAK-STAT signal transduction involved in antiviral and antibacterial responses but also plays a key role in IFN signal transduction and the anti-PD-1/PD-L1 response. We further validated these findings by hMeDIP-qPCR and MeDIP-qPCR analyses. The results showed that the 5hmC level of the IRF1 promoter significantly decreased in TET2-KO cells but increased in vitamin C treated cells, while 5mC presented the opposite trend (Fig. 5B). The methylation status assessed by bisulfite sequencing analysis showed that the methylation level of the IRF1 promoter was increased in TET2-KO cells and hypomethylated after treatment with vitamin C (Fig. 5C).

We also assessed the expression of IRF1 according to the methylation status and found a significant negative correlation in TCGA ccRCC dataset (Fig. 5D). Furthermore, highly methylated IRF1 was negatively correlated with the expression of PD-L1 in TCGA ccRCC tissues (Fig. 5E). We also assessed the correlation of IRF1 and TET2 in ccRCC tissue from TCGA and our data, and we found a significant positive correlation between IRF1 and TET2 (Fig. S5A and S5B). The expression of IRF1 was also associated with PD-L1 in ccRCC tissues from TCGA (Fig. S5C). The expression of TET2 was also associated with PD-L1 in ccRCC tissues (Fig. S5D). Furthermore, the expression of IRF1 was significantly positively associated with CD4+ and CD8+ T cell infiltration, while the promoter methylation level of IRF1 negatively correlated with CD4+ and CD8+ T cell infiltration in ccRCC tissues from TCGA (Fig. S5E and S5F).

**Figure 6:**
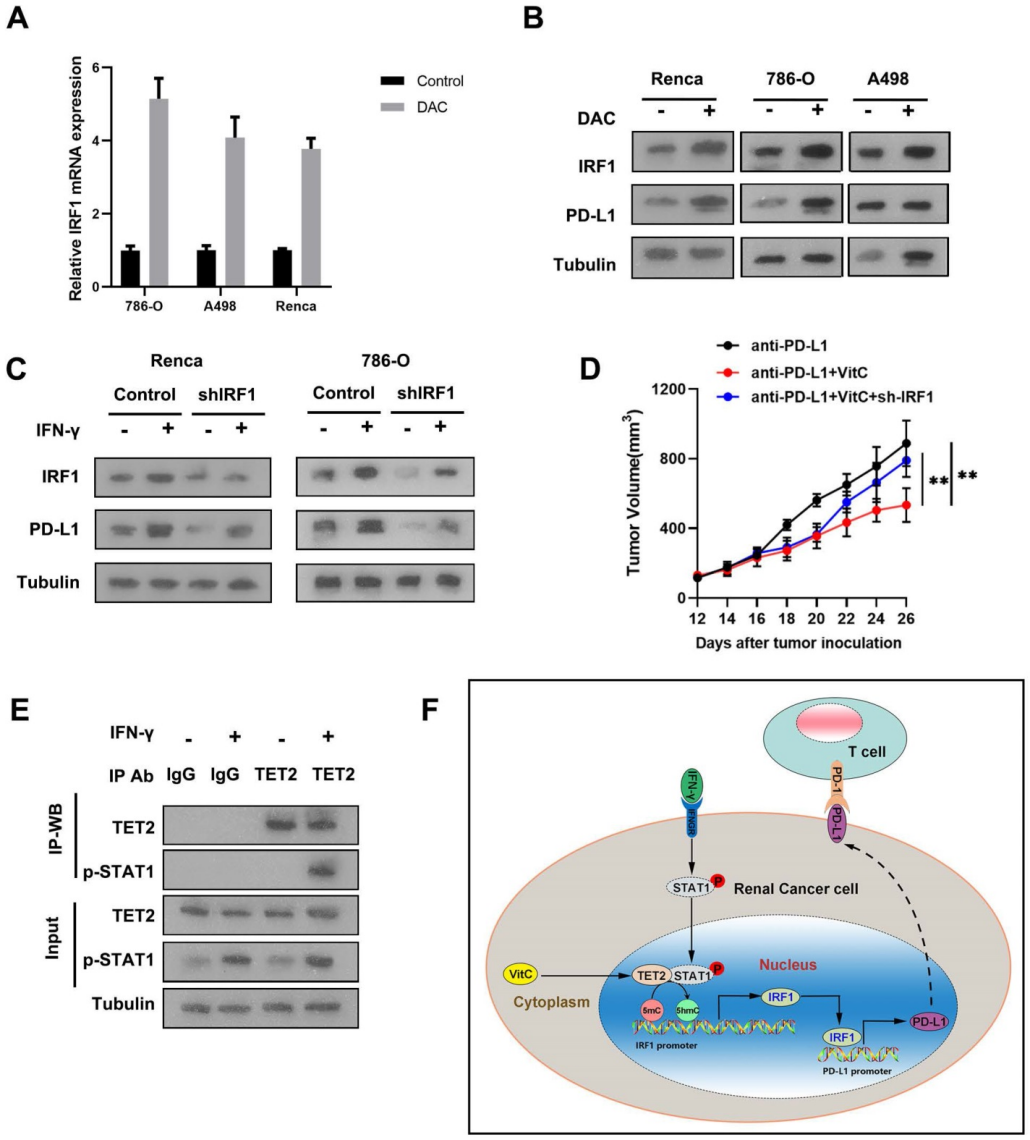
TET2 binds to p-STAT1 to regulate IRF1 demethylation, leading to PD-L1 expression. (A) IRF1 mRNA expression after treatment with DAC or control. (B) IRF1 and PD-L1 protein levels after treatment with DAC or control. (C) Western blot assay of IRF1 and PD-L1 protein levels in sh-IRF1 cells with or without IFN-γ stimulation. (D) Tumor growth of sh-IRF1 Renca cells treated with vitamin C and control cells under treatment with anti-PD-L1 antibody as indicated. (E) Coimmunoprecipitation analysis of TET2 and p-STAT1 in 786-O cells upon IFN-γ stimulation. (F) Schematic of the mechanism by which vitamin C enhances cancer immunotherapy efficacy in renal cell carcinoma via TET2 interaction with IFN-γ activated STAT1 binding to IRF1 to regulate its demethylation and expression, leading to downstream PD-L1 expression. *P < 0.05, **P < 0.01, ***P < 0.001.

To validate the effect of methylation on the expression of IRF1, we treated RCC cells with the methylation inhibitor, decitibine (DAC), and found that the mRNA and protein levels of IRF1 and PD-L1 were elevated after DAC treatment (Fig. 6A and 6B). To further investigate whether the mechanism of the reduced immunotherapy response of TET2-KO tumors relies on decreased IRF1, we knocked down IRF1 in 786-O and Renca cells and found that suppression of IRF1 resulted in decreased PD-L1 expression upon IFN-γ stimulation (Fig. 6C). When comparing the tumor growth of these IRF1 knockdown cells with immunotherapy, IRF1 knockdown reversed the observed combined effect of vitamin C treatment on tumor growth under immunotherapy (Fig. 6D). Thus, these data demonstrated that IRF1 is the main target regulated by TET2 under immunotherapy conditions.

IRF1 is the direct target of IFN-γ-activated STAT1. We further detected that IFN-γ treatment significantly enhanced the interaction of p-STAT1 with TET2, suggesting that TET2 interacts with p-STAT1 after IFN-γ stimulation (Fig. 6E). These findings indicated that IFN-γ-activated STAT1 interacts with TET2 binding to the IRF1 locus to promote IRF1 demethylation and transcription, leading to downstream PD-L1 induction (Fig. 6F).

## Discussion

The present study demonstrated that ascorbic acid enhances the efficacy of immunotherapy and that the loss of TET2 function enables RCC cells to evade antitumor immunity and resist anti-PD-L1 therapy. Vitamin C promoted the enzyme activity of TET2, which is recruited to IRF1 by IFN-γ-STAT1 signaling, thereby maintaining IRF1 demethylation and ultimately inducing PD-L1 expression. Compared to vehicle or single-drug anti-PD-L1 treatment, vitamin C treatment significantly increased the intratumoral infiltration of T cells, while the loss of TET2 impaired the infiltration of T cells.

The PD-1/PD-L1 signaling pathway plays a vital role in tumor immune evasion, but only a fraction of cancer patients respond to immune checkpoint blockade 28. The response to immunotherapy is generally related to inducible PD-L1 expression, and it has been shown that disruption of the IFN-γ pathway leads to resistance to anti-PD-1/PD-L1 therapy 29. Abnormal epigenetic modification patterns and dysregulation of methylcytosine dioxygenase TETs are considered to be hallmarks of cancer, and they have an impact on the development and progression of cancer 11, 30. Genome-wide 5hmC levels have been demonstrated to be downregulated in many cancers, including RCC, and they can be used as a new type of diagnostic and prognostic biomarker 11, 13, 30. As the catalyzing enzymes of 5hmC, TET enzymes act as tumor suppressors in several tumors 12, 31-33. Recent studies have also reported the effect of TET enzymes on immunotherapy. A previous study has shown that in melanoma and colon tumors, both PD-L1 and chemokine genes are silenced by DNA methylation and are activated by TET2-mediated demethylation after inflammation and IFN-γ stimulation 23. Another study has also reported that NAD^+^/α-KG-mediated TET1 synergizes with IFN-γ signaling to regulate PD-L1 expression 21. Another study recently also found yhat TET2 could inhibit PD-L1 gene expression in breast cancer cells through histone deacetylation 34.However, the relationship between TET2 and PD-L1 in renal cancer is still unkown. In the present study, we found that the 5hmC levels of PD-L1 and chemokine genes were not significantly regulated by TET2. Instead, we demonstrated that IFN-γ stimulated TET2 to bind to the promoter of IRF1 and increase the level of 5hmC, thereby promoting the expression of PD-L1.

Ascorbic acid is an essential vitamin for humans due to the evolutionary loss of the gulonolactone oxidase enzyme, which is necessary to catalyze the final step in ascorbic acid synthesis 35. At the same time, the vitamin C level in tumors is also significantly lower than that in normal tissues 36. The anticancer activity of vitamin C may have two main mechanisms as follows: hydrogen peroxide-induced oxidative stress and TET enzyme activation-mediated DNA demethylation. Several studies have previously shown that pharmacological doses of ascorbic acid reduce the growth of aggressive tumors, enhance the cytotoxicity of chemotherapy and radiation therapy and relieve symptoms related to cancer and chemotherapy 23, 37-45. Recent studies have also provided novel insights into the antitumor effects of vitamin C by stimulating chemokine expression and T cell infiltration in solid tumors 19, 20, 23. Another study also reported that high-dose vitamin C synergizes with oncolytic adenoviruses against tumor by inducing ROS and enhances immunogenic tumor cell death and reprogramming tumor immune microenvironment 46. In the present study, we demonstrated that vitamin C stimulated the infiltration of lymphocytes and enhanced the effect of anti-PD-L1 immunotherapy in RCC. Deletion of TET2 substantially reduced the activity of vitamin C to facilitate the survival of RCC cell-bearing mice after injection with anti-PD-L1, suggesting that TET2 is the major target of vitamin C in boosting the antitumor immunity and efficacy of anti-PD-L1 therapy. Thus, we suggest that high-dose vitamin C should be considered as an adjuvant to immunotherapy, especially for solid tumors expressing low levels of 5hmC. An important insight from this finding is that TET activity can be stimulated *in vivo* in solid tumors to achieve therapeutic benefit in response to immunotherapy.

The IFN-γ-STAT1-IRF1 axis plays a vital role in the interaction between tumors and the immune system. In the present study, the changes in the transcriptome profile of tumor cells with dioxygenase deficiency and cofactor activation indicated that the activation of IFN-γ signaling is essential for making tumor cells susceptible to immunotherapy. A previous study has reported that IFN-γ stimulates STAT1 to bind TET2 and recruit TET2 to hydroxymethylate PD-L1 and chemokine genes in melanoma and colon tumor cells 22, 23. However, the present study found that the expression of PD-L1 was regulated by TET2 through the STAT1-IRF1 pathway instead of direct hydroxymethylation regulation. Our results revealed that activation of 5hmC modification sensitizes tumors to immunotherapy by altering the tumor microenvironment and recruitment of CD8+ TILs. This sensitization effect in RCC tumors is mediated by elevated IRF1 and PD-L1. These findings suggest a new strategy by stimulating TET activity to improve immunotherapy for RCC. Thus, vitamin C is reemerging as a potential anticancer agent as a result of a better mechanistic understanding and improved intravenous delivery.

Overall, our work demonstrated that DNA hydroxymethylation plays a vital role in tumor survival during immunotherapy and provides the opportunity to combine immunotherapy with epigenetic agents for RCC therapy.

## Supplementary Material

Supplementary figures and tables.

## Abbreviations

TET: ten-eleven translocation family dioxygenases
5hmC: 5-hydroxymethylcytosine
5mC: 5-methylcytosine
hMeDIP: hydroxymethylated DNA immunoprecipitation
MeDIP: methylated DNA immunoprecipitation
IRF1: interferon regulatory factor-1
PD-L1: programmed death ligand 1
PD-1: programmed death 1
STAT1: signal transducer and activator of transcription 1
IFN-γ: interferon gamma
