# Supplementary material for: Ascorbic acid induced TET2 enzyme activation enhances cancer immunotherapy efficacy in renal cell carcinoma
Source: Int J Biol Sci. 2022 Jan 1;18(3):995–1007. doi: 10.7150/ijbs.67329 (PMC8771844; doi:10.7150/ijbs.67329)
Supplement: Supplementary file 1 — Supplementary figures and tables. [file ijbsv18p0995s1.pdf]

Table1. 255 co-regulated genes significantly altered in RNA-seq analysis

Gene Name	TET2-KO-FC	TET2-KO-pvalue	TET2-KO-padj	VitC-FC	VitC-pvalue	VitC-padj
ACOD1	-1.05312548	4.26E-07	1.44E-05	1.499907	3.20E-06	5.08E-03
AIF1	-2.78507435	5.15E-03	4.42E-02	4.222405	6.13E-03	4.51E-02
CAMK1D	-2.10466129	6.06E-04	8.45E-03	1.081376	0.002596	0.017487329
CCL3	-6.91713866	6.59E-19	2.50E-16	2.461921	0.002596	0.017488347
CCL4	-4.06948566	2.17E-09	1.12E-07	3.152193	5.19E-03	3.50E-02
CCL5	-5.22183265	1.80E-18	1.12E-15	3.842465	7.7E-09	5.2464E-08
CCL8	-8.37417965	1.12E-41	1.27E-38	4.532737	1.04E-10	7.00E-08
CCR2	-7.52652665	1.14E-05	0.00031602	5.223009	1.3E-12	0.00874397
CD180	-6.67887365	9.09E-27	1.95E-13	5.913281	1.56E-09	1.05E-05
CD27	-5.83122065	3.56E-16	1.39E-13	6.603553	1.82E-08	0.001224154
CD274	-4.98356765	1.13E-21	1.27E-12	7.293825	2.08E-07	1.40E-03
CD40	-4.13591465	1.14E-05	0.00016602	7.984097	2.34E-05	0.001573911
CIITA	-1.59295565	4.56E-11	3.38E-09	8.285617	5.02E-17	6.28E-12
CMA1	-1.74530264	6.84E-11	5.07E-09	1.321638	7.53E-17	9.42E-03
CXCL10	-5.51889817	3.48E-05	7.32E-04	4.59804	1.46E-04	2.15E-03
CXCL11	-6.31071168	5.22E-05	0.001	6.28262	2.18E-04	3.22E-03
CXCL12	-7.10252519	6.96E-05	7.32E-04	7.9672	2.91E-04	1.50E-03
CXCL9	-7.8943387	8.70E-05	0.00014	9.65178	3.64E-04	3.33E-03
GBP10	-3.55809626	1.67796E-06	0.00026	6.294296	5E-05	0.005547549
GBP2	-1.62991176	1.53E-03	2.37E-02	2.240446	4.55E-04	5.04E-03
GBP2B	-1.70172727	1.35856E-05	0.000213	3.186597	0.004094	0.045389215
GBP3	-1.77354277	1.22E-06	1.90E-05	4.132748	3.64E-03	4.03E-02
GBP4	-5.84535828	0.000001067	0.00010006	3.078899	0.003184	0.035302945
GBP5	-3.91717379	9.15E-04	1.42E-03	2.02505	2.73E-03	3.03E-02
GBP6	-1.98898929	0.000000076	0.000001	1.9712	0.002274	0.025216675
GBP7	-1.0608048	6.10E-04	9.50E-03	7.917351	1.82E-03	2.02E-02
GBP8	-8.1326203	0.000457626	0.00712396	3.863502	0.001365	0.015130405
GBP9	-6.20443581	3.05E-04	4.75E-03	2.809653	9.10E-04	1.01E-02
IDO1	-4.27625132	0.000152542	0.00237465	2.755804	0.000455	0.005044135
IFI44L	-4.34806682	5.18E-13	4.56E-11	3.701954	2.74E-08	1.00E-06
IFI47	-4.41988233	0.000152542	0.00237465	4.648105	0.000455	0.005042135
IGTP	-4.49169783	3.05E-04	4.75E-03	5.594256	9.10E-04	1.01E-02
IIGP1	-4.56351334	0.000457626	0.00712396	6.540407	0.001365	0.015128405
IL12RB1	-4.63532885	6.10E-04	9.50E-03	7.486558	1.82E-03	2.02E-02
IRF1	-9.13262421	4.57626E-10	7.124E-08	8.211264	1.36E-11	0.000151304
IRF7	-4.20443581	3.05E-05	4.75E-04	5.809653	9.10E-06	1.01E-03
IRGM1	-6.48899125	0.000198304	0.00308701	6.145081	0.000673	0.006479823
IRGM2	-6.83099752	3.87E-04	6.03E-03	5.598326	1.26E-03	1.27E-02
LAT	-2.1730038	0.000576608	0.00897614	4.05157	0.00185	0.018955677
MCPT4	-3.51501008	7.66E-04	1.19E-02	4.504814	2.44E-03	2.52E-02
MEFV	-4.85701635	0.000954913	0.01486526	4.958059	0.003028	0.031431531

NOS2	-5.54102891	0.001333217	0.02075439	5.864548	0.00042	0.004390739
OAS3	-2.88303519	1.52E-03	2.37E-02	6.317792	4.79E-04	5.01E-03
OASL2	-2.22504146	0.001711521	0.02664351	2.771036	5.38E-05	0.005638324
PDCD1	-3.56704774	1.90E-03	2.96E-02	3.224281	5.97E-05	6.26E-03
PTAFR	-2.90905402	0.002089825	0.03253264	2.677525	6.56E-05	0.006885909
STAT1	-4.2510603	2.28E-03	3.55E-02	3.130769	7.15E-05	7.51E-03
STAT4	-5.59306657	0.002468129	0.03842176	3.584014	7.74E-07	0.008133495
TGTP1	-6.93507285	2.66E-03	4.14E-02	4.037258	8.32E-05	8.76E-03
TGTP2	-8.27707913	0.002846433	0.04431089	2.490503	8.91E-08	0.000938108
THEMIS2	-9.61908541	3.04E-03	4.93E-02	2.943747	9.50E-07	1.00E-04
TLR12	-2.96109168	0.000322474	0.00502	3.396991	1.01E-07	0.000106287
TLR9	-2.30309796	3.41E-04	5.31E-03	4.850236	1.07E-05	1.13E-03
ABCC2	-1.92029892	1.08E-10	7.05E-09	1.132868	2.90E-05	0.000485878
ACACB	-1.4867122	2.13E-07	7.22E-06	1.316387	1.29E-05	0.000242685
ADAMTS2	-3.30827993	1.66E-09	8.68E-08	1.568536	0.000507	0.005543964
ADGRB2	-2.54410706	1.03E-05	0.00023197	2.133719	0.000401	0.004573045
AFAP1	-1.50933692	2.19E-13	2.09E-11	1.036195	1.06E-08	4.18E-07
AFF2	-4.35208086	9.06E-06	0.00020657	7.691226	1.67E-06	4.06E-05
AGXT2	-1.75545401	0.0038587	0.03312291	1.762095	0.004594	0.033786855
AIF1L	-1.79920643	3.00E-28	1.39E-25	3.669575	3.50E-78	8.31E-75
AJAP1	-8.56959931	6.59E-09	3.07E-07	9.909956	2.20E-11	1.38E-09
ALOX5AP	-2.84808124	1.36E-17	2.28E-15	2.955884	1.29E-17	1.71E-15
ALPK3	-8.04710463	1.41E-14	1.65E-12	4.854597	1.75E-113	5.80E-110
AMPH	-9.79751479	4.18E-11	2.92E-09	8.252286	2.28E-08	8.45E-07
AOX1	-1.41761855	3.19E-05	0.000611	1.398124	4.58E-05	0.000721178
APOBEC3G	-4.73201477	4.25E-10	2.50E-08	2.458421	3.25E-06	7.30E-05
ARHGDIB	-2.61710758	1.31E-10	8.42E-09	1.049748	0.002973	0.023988134
ARTN	-3.04707048	1.39E-37	1.03E-34	1.888862	1.29E-16	1.51E-14
ASS1	-9.71314838	2.75E-11	2.00E-09	9.610943	3.51E-11	2.11E-09
AUTS2	-6.37568048	0.000458788	0.00598096	6.273432	0.000638	0.006767625
BAHCC1	-6.09732488	5.91E-14	6.01E-12	1.22334	0.000481	0.005306417
BARX2	-6.38001693	5.66E-05	0.00100812	6.279514	7.37E-05	0.00108284
BCO1	-1.49914048	0.001365306	0.01462317	1.653747	0.001025	0.010100311
BGN	-8.06533671	2.73E-07	9.04E-06	4.191558	2.51E-06	5.76E-05
BMP7	-6.86529212	6.63E-05	0.00115575	5.323702	0.001854	0.016272054
C1QTNF1	-1.20546572	6.62E-11	4.48E-09	3.781094	2.59E-52	2.39E-49
C2CD4C	-3.85797667	2.09E-07	7.12E-06	1.676953	0.003447	0.027032615
C6orf141	-6.48131898	0.00030301	0.00422614	6.379165	0.000426	0.004772643
CCDC88C	-1.88593232	1.28E-09	6.90E-08	1.771648	1.32E-08	5.09E-07
CDIP1	-3.28826165	3.44E-54	4.55E-51	2.213576	3.97E-35	1.47E-32
CIART	-2.44060865	2.28E-11	1.69E-09	5.249597	2.51E-17	3.14E-15
COCH	-6.90702343	5.96E-05	0.00104971	6.805028	8.62E-05	0.00125154
COL12A1	-3.17207118	1.61E-19	3.42E-17	2.081277	4.10E-11	2.43E-09
COL13A1	-4.81573446	0.000219713	0.00323027	2.396196	0.007443	0.049385884

COL14A1	-2.36866931	7.59E-19	1.50E-16	1.804079	1.37E-12	9.98E-11
COL5A2	-1.87283596	2.87E-09	1.42E-07	2.159081	2.79E-10	1.43E-08
COL6A3	-5.18770508	1.23E-208	2.11E-204	6.182437	7.83E-194	6.51E-190
COL9A2	-3.47307768	3.91E-05	0.00072874	1.931943	0.005347	0.037955516
CORO2B	-4.69110205	5.80E-18	1.02E-15	3.309039	2.88E-13	2.30E-11
CPE	-4.27287308	9.09E-67	1.95E-63	2.968702	6.08E-46	3.74E-43
CRACR2B	-2.78923967	3.56E-26	1.39E-23	2.321531	2.65E-20	4.79E-18
CRIP2	-1.93527115	1.23E-41	1.17E-38	1.22888	4.39E-19	6.76E-17
CXADR	-6.72708466	1.74E-05	0.00036602	2.91346	7.28E-05	0.00107455
CYBRD1	-6.42503855	0.000383453	0.00512734	4.884056	0.006441	0.043950261
DAAM2	-6.12864547	0.000139598	0.00221103	4.445648	5.18E-05	0.00080476
DENND2D	-6.90082858	7.84E-06	0.0001821	6.797787	1.03E-05	0.000199749
DEPP1	-1.17653042	1.57E-23	5.29E-21	1.308659	9.51E-28	2.59E-25
DGKA	-5.52057735	0.001069047	0.01198072	6.860467	6.26E-05	0.000944701
DHRS9	-2.53199304	1.13E-08	5.05E-07	2.607829	3.54E-09	1.52E-07
DSP	-7.80766038	3.59E-19	7.43E-17	10.73329	2.52E-13	2.03E-11
DYSF	-5.65545802	1.17E-30	6.50E-28	1.111073	2.91E-06	6.61E-05
EDN2	-2.73098726	1.48E-11	1.12E-09	4.123504	1.12E-15	1.15E-13
EFHD1	-6.93249199	7.10E-06	0.00016658	3.36922	1.00E-06	2.56E-05
ENPP3	-2.32981258	4.83E-23	1.53E-20	1.781138	1.41E-18	1.98E-16
EPB41L3	-7.32615849	8.69E-06	0.00019948	7.223956	1.30E-05	0.00024474
EPHX4	-7.2748694	1.09E-05	0.00024468	5.732645	0.000497	0.00545091
FAM20C	-1.52716998	4.22E-22	1.21E-19	1.156551	3.43E-14	2.99E-12
FAM43B	-5.51937169	0.001069911	0.01198258	6.860467	6.26E-05	0.000944701
FCHO1	-3.33691364	1.68E-05	0.00035679	3.550301	9.15E-06	0.000180939
FHDC1	-1.38286852	0.000659957	0.00811497	1.196076	0.002568	0.021204459
FYN	-2.3363939	3.83E-05	0.00071647	2.420873	3.95E-05	0.000636002
GALNT5	-10.9981849	5.87E-14	6.01E-12	8.452767	8.67E-16	9.12E-14
GALNT9	-1.61235585	8.90E-16	1.15E-13	1.030693	6.35E-08	2.15E-06
GFRA2	-4.27625132	0.000152542	0.00237465	5.755804	0.000455	0.005044135
GJA1	-2.34806682	5.18E-13	4.56E-11	1.701954	2.74E-08	1.00E-06
GPX3	-1.70270807	3.03E-11	2.18E-09	1.052404	1.23E-05	0.000233628
GRAMD1B	-6.6071251	0.000196859	0.00294928	4.075266	0.004876	0.035364096
GTF2IP1	-2.21411736	1.56E-19	3.36E-17	1.595391	1.60E-11	1.02E-09
HHIPL2	-1.39771674	0.000290495	0.00406812	1.810063	5.50E-06	0.000114553
HMGA1	-1.80579575	5.51E-10	3.15E-08	1.712891	5.96E-09	2.44E-07
HNMT	-7.61066583	2.98E-06	7.71E-05	6.068353	0.000183	0.002396413
HS6ST2	-9.2330699	7.83E-10	4.37E-08	5.694391	1.25E-11	8.14E-10
HTR7	-2.31448023	0.002927958	0.02681073	2.322902	0.0028	0.022770238
IGF2	-6.41748442	4.93E-05	0.00089291	7.757678	1.13E-06	2.86E-05
IGFBP1	-2.53990112	3.98E-22	1.16E-19	5.925869	6.94E-36	2.81E-33
IGFBP4	-9.46745496	2.29E-10	1.41E-08	9.365274	3.78E-10	1.91E-08
IL10RA	-4.35041043	0.000135009	0.0021423	3.245983	0.000585	0.006274734
IL11	-2.85026552	1.27E-09	6.86E-08	1.54878	0.000123	0.001694553

INHBB	-3.49838834	9.45E-58	1.47E-54	3.669408	1.70E-58	2.17E-55
INPP4B	-2.81147613	4.06E-06	0.00010149	2.972782	2.95E-06	6.70E-05
IRF8	-2.32731579	0.004506669	0.03765374	4.80316	0.000194	0.002513239
ITGB7	-5.08372859	0.003888047	0.03324397	4.986108	0.004828	0.035118992
ITIH5	-3.23864875	3.30E-27	1.38E-24	4.740405	8.36E-34	3.02E-31
KCNIP3	-5.26371658	3.51E-19	7.34E-17	5.006093	2.78E-18	3.78E-16
KCNJ16	-2.22552237	7.15E-07	2.15E-05	1.027354	7.71E-08	2.52E-06
KCNK3	-1.24830638	5.26E-09	2.49E-07	1.351054	8.61E-10	4.10E-08
KRT19	-10.5393093	1.02E-10	6.66E-09	7.69472	5.26E-198	8.74E-194
KRT79	-1.30513292	7.63E-07	2.28E-05	2.667366	2.10E-15	2.08E-13
KSR1	-2.35566069	0.000461705	0.00600987	3.426024	1.66E-05	0.000298805
LAMB3	-1.36467033	6.22E-34	4.45E-31	1.409413	1.39E-37	6.25E-35
LAYN	-10.4139463	1.88E-12	1.61E-10	7.281681	4.04E-16	4.50E-14
LCN2	-1.00005112	0.002629601	0.02456345	2.382116	4.55E-09	1.92E-07
LIMS2	-4.00018733	6.78E-09	3.14E-07	3.605279	5.34E-08	1.84E-06
LINC00462	-5.5886251	0.000856825	0.0100312	6.929739	4.65E-05	0.000729896
LINC00528	-5.1361911	0.003403973	0.03009414	3.444772	0.007292	0.04856386
LINC01929	-2.90497675	2.72E-05	0.000532	4.920995	2.01E-06	4.80E-05
LIPG	-2.26356352	1.75E-05	0.00036779	5.068448	9.97E-09	3.94E-07
LOC105369306	-7.16116666	1.82E-05	0.00037754	7.059039	2.69E-05	0.000454657
LONRF3	-4.35983485	5.00E-08	1.92E-06	6.843249	9.00E-06	0.000178276
LOX	-3.42113346	1.92E-16	2.72E-14	3.274171	4.07E-15	3.95E-13
LOXL1	-2.48754398	2.96E-12	2.48E-10	6.385501	1.24E-15	1.26E-13
LOXL1-AS1	-6.41241193	5.04E-05	0.00090827	7.753234	1.17E-06	2.93E-05
LRRC15	-3.80871726	2.71E-06	7.13E-05	1.824002	0.00499	0.036013553
LRRC34	-5.05723599	4.11E-10	2.43E-08	3.698096	5.57E-09	2.31E-07
LTBP4	-3.12453581	4.53E-103	2.59E-99	3.685768	3.38E-123	1.40E-119
MATN2	-2.82930057	6.12E-14	6.18E-12	1.222639	0.000115	0.001591944
ME3	-3.15955865	0.000479229	0.00619103	4.282211	0.000139	0.00189088
MFNG	-2.42621927	7.13E-21	1.78E-18	1.512776	7.37E-11	4.14E-09
MLPH	-5.09071036	0.003862314	0.03312291	4.001229	0.006077	0.041968135
MMP13	-3.50756691	2.24E-09	1.15E-07	4.732816	4.89E-10	2.43E-08
MMP2	-3.23677312	2.33E-67	5.73E-64	1.239877	3.34E-14	2.92E-12
MT1L	-6.75857374	9.89E-10	5.42E-08	7.655246	3.15E-07	9.14E-06
MX1	-1.7710678	8.57E-24	2.94E-21	1.132194	1.15E-10	6.31E-09
NES	-5.0840736	6.17E-07	1.89E-05	4.572581	1.15E-06	2.90E-05
NID1	-10.425939	7.27E-13	6.37E-11	7.000405	2.43E-45	1.44E-42
NKX2-5	-6.62527607	0.000177823	0.0027066	6.52322	0.000252	0.00312119
NLRP3	-5.03746478	1.16E-06	3.31E-05	4.925359	2.32E-06	5.43E-05
NOG	-8.74554415	9.53E-09	4.31E-07	7.20199	2.05E-06	4.90E-05
NRG2	-2.26137814	0.000798802	0.00947797	3.006639	8.96E-05	0.001289267
NTN1	-2.45490634	0.002150472	0.02089499	3.354427	0.000289	0.003494155
NTRK3	-4.27069978	1.16E-07	4.08E-06	3.756388	6.09E-07	1.64E-05
OAF	-1.13936425	1.37E-18	2.61E-16	1.074248	1.81E-17	2.31E-15

OPLAH	-2.94285374	9.42E-05	0.00156417	4.293734	1.09E-05	0.000211181
OSBPL1A	-9.56514879	1.41E-10	8.91E-09	9.463021	2.32E-10	1.22E-08
PAPLN	-1.3259617	6.39E-09	2.99E-07	5.457021	1.03E-13	8.65E-12
PAQR7	-2.28078495	3.54E-05	0.00066843	3.027313	1.36E-06	3.36E-05
PCDH1	-2.42865271	3.48E-07	1.12E-05	4.286432	4.86E-11	2.85E-09
PDGFRL	-1.93635331	0.000171339	0.00261952	5.012155	2.83E-08	1.03E-06
PDPN	-7.02376242	5.09E-05	0.00091581	6.921923	7.34E-05	0.001080246
PGF	-2.06157175	0.000344376	0.00470374	1.714676	0.002289	0.019300061
PHETA2	-6.99638357	5.44E-06	0.0001309	3.716133	3.42E-07	9.82E-06
PIK3AP1	-5.20673035	3.00E-11	2.17E-09	2.066762	9.87E-06	0.000192992
PKP2	-3.44285776	1.29E-11	9.89E-10	4.665383	5.50E-15	5.17E-13
PLCG2	-5.09322615	9.59E-09	4.32E-07	8.432854	4.41E-08	1.55E-06
PLXNA2	-1.75160858	1.15E-05	0.00025636	6.219849	2.28E-11	1.42E-09
PNMA2	-2.09058463	0.002723791	0.02525095	4.646601	1.38E-05	0.000257723
POGLUT3	-11.1256778	2.63E-14	2.97E-12	5.673922	1.65E-38	7.85E-36
POU3F3	-8.42725273	4.74E-08	1.84E-06	8.325021	7.50E-08	2.47E-06
PPFIBP2	-2.61311216	4.04E-06	0.0001012	1.382919	0.005338	0.037953173
PPM1M	-1.39867673	0.000156478	0.00242252	1.61631	3.02E-05	0.000503801
PRTFDC1	-7.622975	4.41E-07	1.40E-05	8.96319	3.31E-09	1.43E-07
PRUNE2	-3.1199763	0.000115893	0.00187361	2.726925	0.000523	0.00569883
PRXL2A	-7.00961973	3.77E-05	0.0007065	6.907599	5.50E-05	0.000845433
PTGES	-1.24313517	1.54E-06	4.26E-05	3.050443	6.04E-20	1.07E-17
PTHLH	-2.48510176	1.73E-27	7.44E-25	2.04931	1.51E-20	2.76E-18
PTK7	-6.92677521	5.02E-05	0.00090576	4.38218	0.001181	0.011302787
PTPRU	-2.61032518	6.41E-17	9.83E-15	3.408384	8.45E-22	1.87E-19
PXDN	-8.02085287	3.47E-14	3.79E-12	6.916013	5.05E-19	7.70E-17
RBM20	-4.0363544	2.00E-05	0.00040822	3.253378	6.99E-05	0.001035023
RELN	-2.38698811	3.87E-16	5.24E-14	7.05416	8.04E-20	1.39E-17
RYR2	-3.05146555	0.000948891	0.01085377	4.758621	0.000218	0.00277354
SARDH	-8.86367729	5.43E-09	2.57E-07	3.622085	3.08E-09	1.34E-07
SATB1	-7.21662578	3.17E-11	2.26E-09	5.795746	1.08E-14	9.86E-13
SCARF2	-5.51597056	2.85E-38	2.33E-35	1.766287	5.69E-12	3.87E-10
SCD5	-6.6278619	0.00018084	0.00274536	6.525843	0.000256	0.003158959
SCN8A	-8.44004758	4.43E-08	1.72E-06	2.994741	3.02E-06	6.85E-05
SDC3	-1.50891056	3.88E-27	1.59E-24	2.499318	1.91E-65	2.88E-62
SEMA6D	-3.15074737	1.33E-10	8.47E-09	1.028539	0.007138	0.047706518
SERPINB9	-6.15385095	0.000127902	0.00203516	3.466447	0.000129	0.001768344
SFMBT2	-5.2283561	0.002696057	0.02503439	5.130703	0.003365	0.026512263
SLAMF7	-7.48274066	4.29E-06	0.00010645	7.380608	6.49E-06	0.000132596
SLAMF8	-5.02926993	0.004604487	0.03830157	6.370477	0.000452	0.005017162
SLC38A5	-3.86032146	8.46E-12	6.60E-10	4.248109	1.44E-11	9.29E-10
SLC4A8	-5.15021586	8.81E-11	5.84E-09	3.784778	1.01E-09	4.76E-08
SLC6A13	-5.27712174	3.45E-07	1.11E-05	2.123533	0.000945	0.009423442
SLC7A2	-8.64560701	4.64E-09	2.22E-07	4.539487	6.76E-21	1.31E-18

SLFN13	-2.17571591	3.21E-08	1.28E-06	3.390206	2.19E-13	1.79E-11
SOAT2	-1.76254866	0.000260873	0.00372623	2.572631	3.76E-06	8.28E-05
SORCS2	-5.43871587	1.51E-59	2.89E-56	3.039183	1.92E-40	9.98E-38
SOX13	-1.55446564	0.000357359	0.00483872	1.708308	7.96E-05	0.0011625
SP5	-7.60236561	4.57E-07	1.44E-05	2.855405	1.84E-08	6.95E-07
SPINT1	-8.07326904	2.83E-07	9.29E-06	4.934903	2.48E-06	5.72E-05
SPN	-8.04871909	6.59E-08	2.44E-06	4.626993	5.75E-15	5.37E-13
SRPX	-1.72913775	3.34E-09	1.63E-07	1.304195	4.06E-06	8.86E-05
STAT5A	-4.49643476	0.000946652	0.01083538	6.839328	7.34E-05	0.00108036
SULT1A1	-4.89144916	5.43E-11	3.70E-09	3.679343	1.83E-09	8.26E-08
SYT12	-4.47376803	4.71E-14	4.96E-12	3.585852	1.94E-12	1.39E-10
TCEA3	-3.37515519	3.97E-14	4.27E-12	4.853539	2.24E-15	2.21E-13
TESC	-6.91834763	5.74E-05	0.00101936	6.815989	8.32E-05	0.001212577
TFCP2L1	-2.73038867	0.000526174	0.00672665	3.514648	0.000146	0.001973514
TGFBR3L	-2.12044564	0.003432444	0.03022135	2.187215	0.005016	0.036142119
TMEM92	-3.15046115	5.37E-12	4.35E-10	1.301655	6.29E-05	0.000948518
TMEM98	-6.38733312	4.30E-22	1.21E-19	8.869538	1.41E-09	6.47E-08
TNF	-2.41856503	0.000472337	0.00612042	2.494394	0.000242	0.003015173
TNFRSF9	-3.30399934	6.39E-58	1.10E-54	3.422998	4.09E-58	4.85E-55
TNS1	-1.02804489	5.73E-18	1.01E-15	1.16492	1.44E-22	3.32E-20
TP53INP2	-2.16149211	1.35E-19	2.96E-17	1.981023	4.57E-16	4.99E-14
TRIM55	-2.3205133	1.08E-37	8.46E-35	1.457926	9.81E-19	1.42E-16
TRPM2	-1.05632446	4.05E-10	2.40E-08	1.161995	1.30E-11	8.47E-10
TSPAN15	-2.11258645	1.42E-08	6.17E-07	3.363965	3.94E-15	3.85E-13
TSPAN18	-8.96845492	3.20E-09	1.57E-07	8.866201	5.18E-09	2.17E-07
TXNIP	-1.20451989	5.90E-12	4.75E-10	2.631449	8.69E-36	3.44E-33
TYMP	-2.82487912	9.15E-22	2.45E-19	1.072892	4.97E-06	0.000105224
ULK2	-9.92798026	2.01E-11	1.51E-09	4.297976	2.99E-19	4.69E-17
UNC13C	-2.21520133	8.62E-06	0.00019805	1.508603	0.001497	0.013764657
UNC5B	-7.69934144	1.78E-51	2.19E-48	4.17093	3.31E-75	6.87E-72
VAMP5	-3.72536841	1.03E-21	2.71E-19	4.54007	3.85E-22	8.76E-20
VDR	-1.44776552	0.001404308	0.01493731	1.499308	0.00109	0.010619875
WASF3	-3.95668871	7.59E-23	2.37E-20	1.401605	1.07E-06	2.71E-05
WNT7B	-2.37413954	1.64E-17	2.73E-15	3.705081	2.89E-26	7.38E-24
YPEL3	-1.6701073	0.000584576	0.00733133	1.801746	0.000367	0.004251544
ZDHHC11	-1.43535689	0.001847799	0.01838057	2.051121	6.86E-05	0.001023818
ZNF185	-1.17729622	0.000448426	0.00585478	1.761153	1.97E-06	4.72E-05
ZNF22	-9.68585819	7.27E-11	4.87E-09	8.141817	3.65E-08	1.30E-06

Table2. 42 overlapping genes from both hMeDIP-seq and RNA-seq data.

SYMBOL	ENSEMBL	ENTREZID
AFAP1	ENSG00000196526	60312
AGXT2	ENSG00000113492	64902
AIF1	ENSG00000204472	199
AJAP1	ENSG00000196581	55966
AMPH	ENSG00000078053	273
ASS1	ENSG00000130707	445
BCO1	ENSG00000135697	53630
BMP7	ENSG00000101144	655
C1QTNF1	ENSG00000173918	114897
CIITA	ENSG00000179583	4261
CMA1	ENSG00000092009	1215
COL6A3	ENSG00000163359	1293
COL9A2	ENSG00000049089	1298
CPE	ENSG00000109472	1363
DYSF	ENSG00000135636	8291
FAM20C	ENSG00000177706	56975
GALNT9	ENSG00000182870	50614
GBP2	ENSG00000162645	2634
GFRA2	ENSG00000168546	2675
HTR7	ENSG00000148680	3363
IDO1	ENSG00000131203	3620
IGF2	ENSG00000167244	3481
IRF1	ENSG00000125347	3659
IRF7	ENSG00000185507	3665
KCNIP3	ENSG00000115041	30818
LRRC15	ENSG00000172061	131578
MEFV	ENSG00000103313	4210
MLPH	ENSG00000115648	79083
MMP2	ENSG00000087245	4313
NRG2	ENSG00000158458	9542
NTN1	ENSG00000065320	9423
OSBPL1A	ENSG00000141447	114876
PIK3AP1	ENSG00000155629	118788
PLCG2	ENSG00000197943	5336
PPFIBP2	ENSG00000166387	8495
PTPRU	ENSG00000060656	10076
RELN	ENSG00000189056	5649
SLC7A2	ENSG00000003989	6542
TNS1	ENSG00000079308	7145
TSPAN18	ENSG00000157570	90139
WASF3	ENSG00000132970	10810
WNT7B	ENSG00000188064	7477

Supplementary Figures:

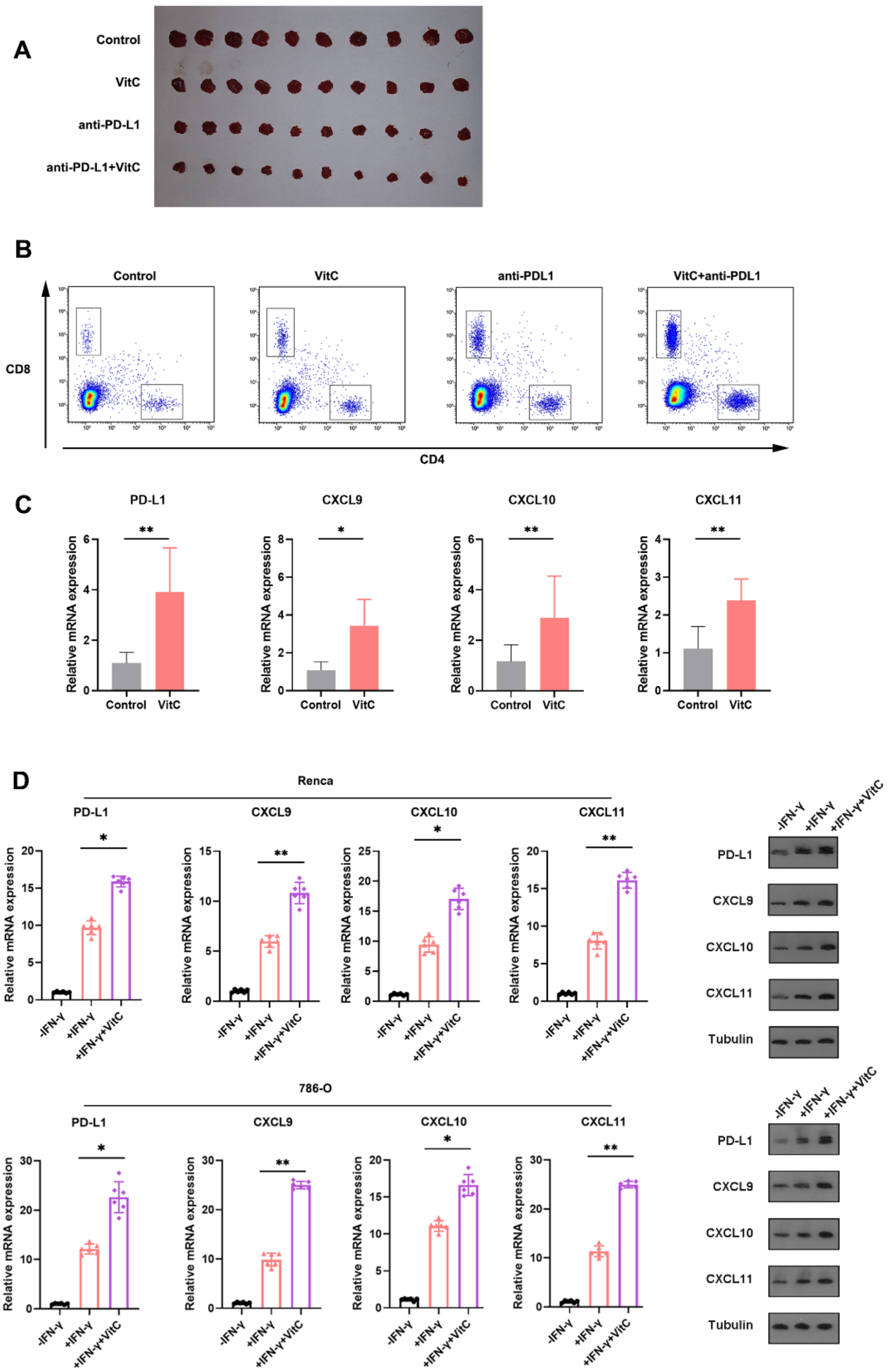

Fig. S1 (A) The images of transplanted tumor in Figure 1B. (B) CD4+ and CD8+ T cells in tumor lysates detected by flow cytometry from tumors in Figure 1B. (C) Expression of the PD-L1, CXCL9, CXCL10 and CXCL11 chemokines in Renca allograft tumors treated with vehicle and vitamin C. (D) Expression of the PD-L1,

CXCL9, CXCL10 and CXCL11 chemokines in Renca and 786-O cells treated with IFN-γ and vitamin C. *P < 0.05, **P < 0.01, ***P < 0.001.

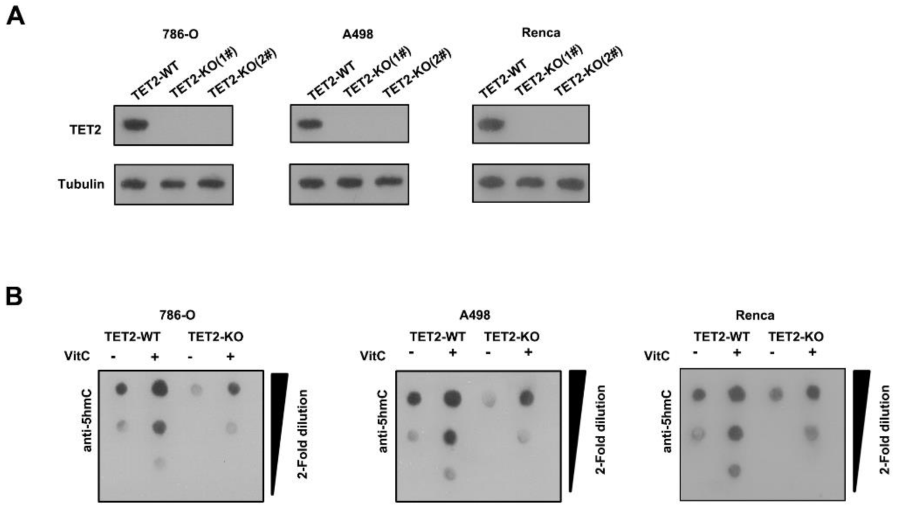

Fig. S2 (A) Western blot assays of TET2 knockout efficiency in the selected single-cell clones. (B) Dot blot assays for 5hmC treated with or without vitamin C in TET2 knockout 786-O, A498 and Renca cells.

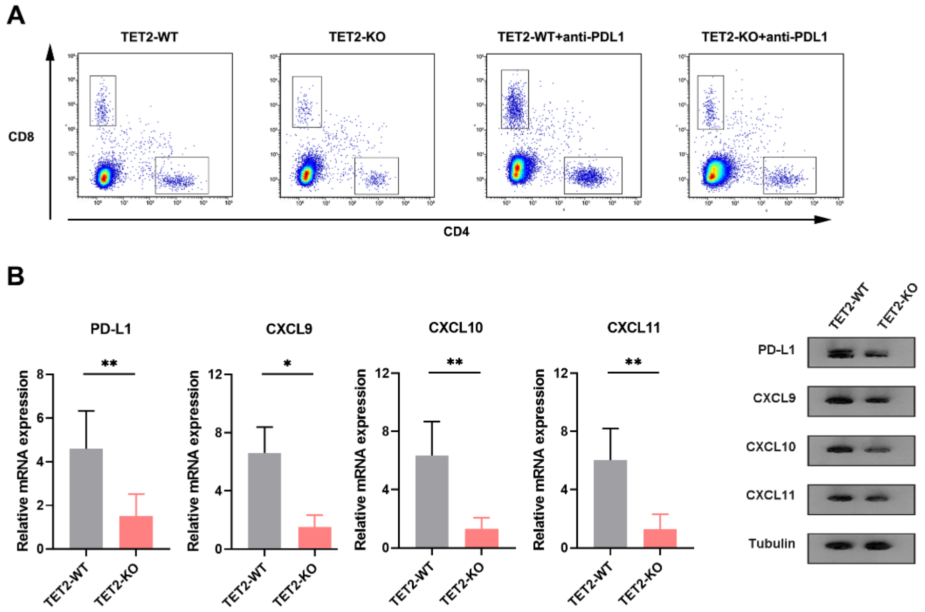

Fig. S3 (A) CD4+ and CD8+ T cells in tumor lysates detected by flow cytometry from tumors in Figure 3A. (B) Expression of the PD-L1, CXCL9, CXCL10 and CXCL11

chemokines in TET2-WT or TET2-KO Renca allograft tumors. *P < 0.05, **P < 0.01, ***P < 0.001.

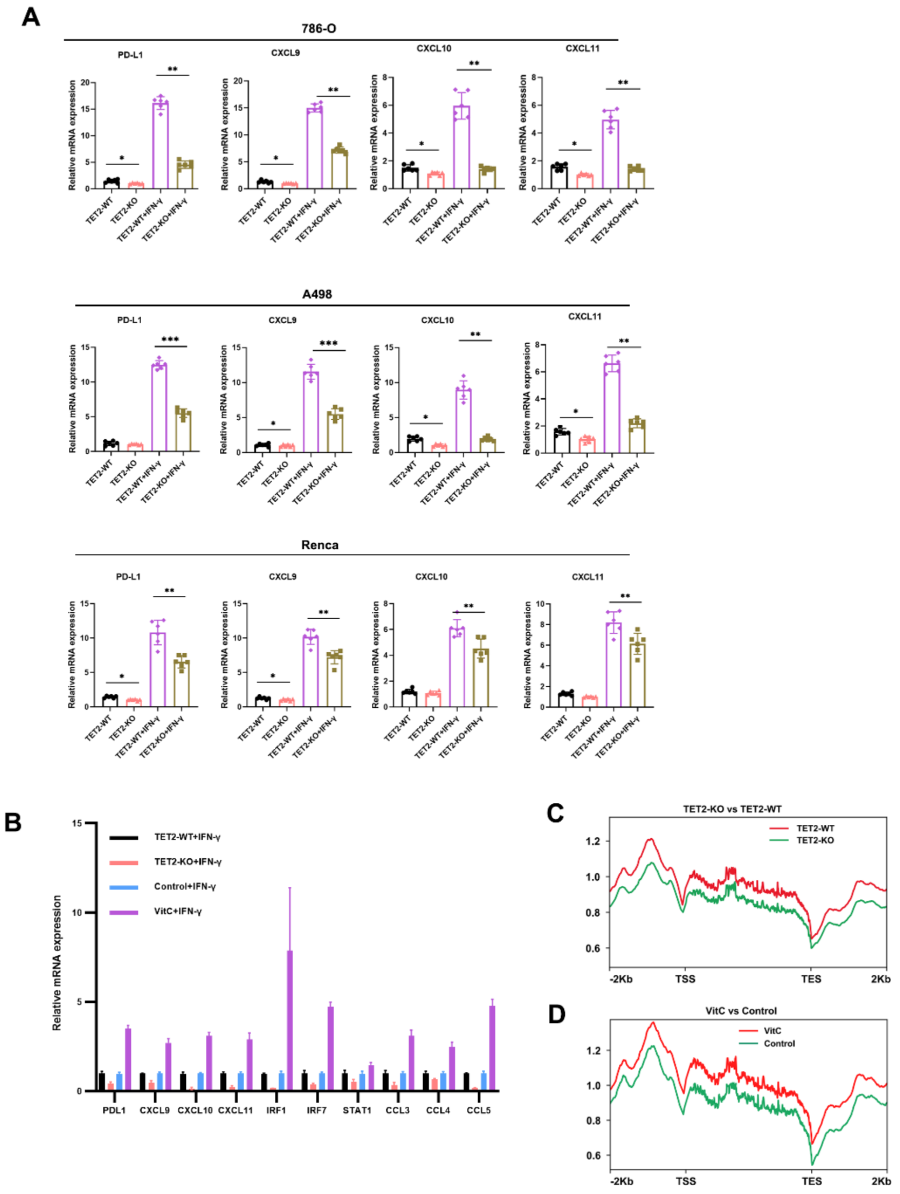

Fig. S4 (A) Expression of the PD-L1, CXCL9, CXCL10 and CXCL11 chemokines in TET2-WT or TET2-KO cells treated with or without IFN-γ. (B) Transcriptional analysis of the indicated genes identified from the RNA-seq analysis using qRT–PCR. (C) Average 5hmC levels in TET2-WT or TET2-KO cells across different gene-associated regions. (D) Average 5hmC levels after treatment with vitamin C or control across different gene-associated regions. *P < 0.05, **P < 0.01, ***P < 0.001.

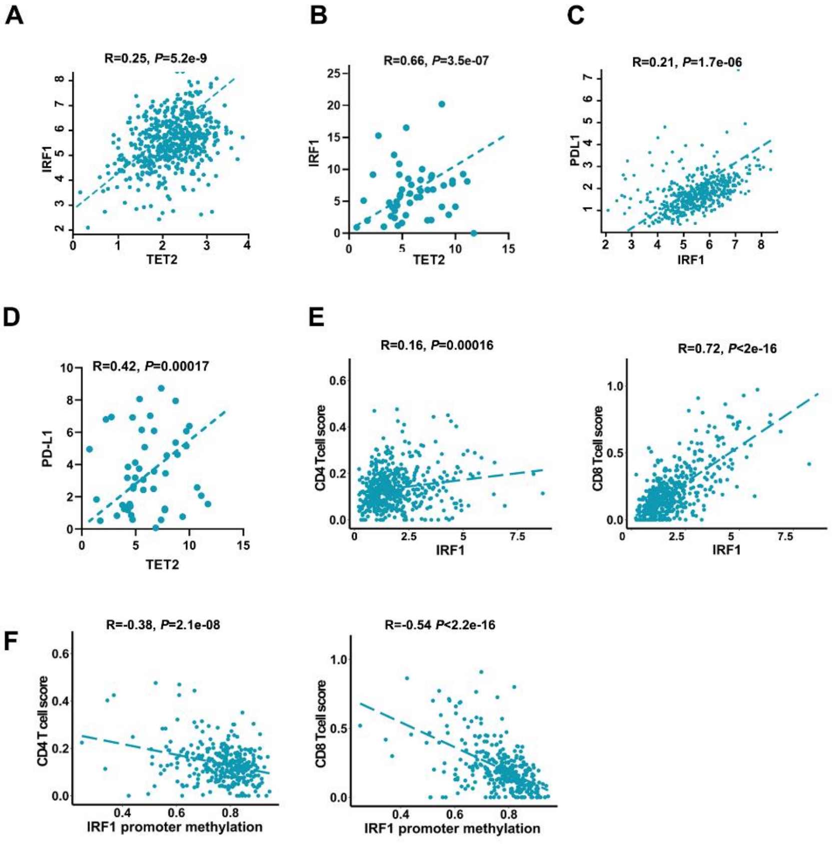

Fig. S5 (A) The correlation of the expression of IRF1 and TET2 in ccRCC tissues from TCGA. (B) The correlation of IRF1 and TET2 in ccRCC tissues from our qRT–PCR data. (C) The correlation of IRF1 and PD-L1 in ccRCC tissues from TCGA. (D) The correlation of PD-L1 and TET2 in ccRCC tissues from our qRT–PCR data. (E) The correlation of CD4+ and CD8+ T cell infiltration with the mRNA expression of IRF1 in ccRCC tissues from TCGA. (F) The correlation of CD4+ and CD8+ T cell infiltration with the promoter methylation level of IRF1 in ccRCC tissues from TCGA.
